# Predictors of CPAP failure after less-invasive surfactant administration in preterm infants

**DOI:** 10.3389/fped.2024.1444906

**Published:** 2024-08-27

**Authors:** Miguel Alsina-Casanova, Nerea Brito, Carla Balcells-Esponera, Ana Herranz-Barbero, Marta Teresa-Palacio, Aleix Soler-García, Carmen Agustí, Guillem Brullas, Jordi Clotet, Cristina Carrasco, Dolors Salvia, Victoria Aldecoa-Bilbao

**Affiliations:** ^1^ Neonatology Department, Hospital Clínic Barcelona, BCNatal (Barcelona Center for Maternal-Fetal and Neonatal Medicine), Barcelona, Spain; ^2^ Neonatology Department, Hospital Sant Joan de Déu, BCNatal (Barcelona Center for Maternal-Fetal and Neonatal Medicine), Barcelona, Spain; ^3^ Pediatrics Department, Hospital Sant Joan de Déu, Barcelona, Spain

**Keywords:** CPAP failure, less-invasive surfactant administration, lung ultrasound, neonatal respiratory distress syndrome, preterm infant

## Abstract

**Introduction:**

Less-invasive surfactant administration (LISA) is associated with better respiratory outcomes in preterm infants with respiratory distress syndrome. However, mechanical ventilation (MV) shortly after the LISA procedure has been related to lower survival. This study aimed to analyze the trends and main predictors of continuous positive airway pressure (CPAP) failure after LISA.

**Material and methods:**

Preterm infants born between 23^0^ and 33^6^ weeks gestational age (GA) in two level III neonatal units who received surfactant were included (2017–2022). Demographic data, lung ultrasound (LUS) scores, the saturation/fraction of inspired oxygen (SF) ratio, technique, time to surfactant administration, and the main neonatal outcomes were collected.

**Results:**

Over the study period, 289 inborn preterm infants received surfactant, 174 with the LISA method (60.2%). Patients who received surfactant after intubation in the delivery room (*n* = 56) were more immature and exhibited worse outcomes. Patients who received surfactant via an endotracheal tube in the neonatal intensive care unit (*n* = 59) had higher LUS scores and a lower SF ratio than those treated with LISA. The LISA method was associated with less death or bronchopulmonary dysplasia (BPD), with an adjusted odds ratio (aOR) = 0.37 [95% confidence interval (CI), 0.18–0.74, *p* = 0.006]. CPAP failure after LISA (defined as the need for intubation and MV in the first 72 h of life) occurred in 38 patients (21.8%), inversely proportional to GA (38.7% at 23–26 weeks, 26.3% at 27–30 weeks, and 7.9% at 30–33 weeks (*p* < 0.001). CPAP failure after LISA was significantly related to death, with an aOR = 12.0 (95% CI, 3.0–47.8, *p* < 0.001), and moderate to severe BPD, with an aOR = 2.9 (95% CI, 1.1–8.0, *p* = 0.035), when adjusting for GA. The best predictors of CPAP failure after LISA were GA, intrauterine growth restriction, temperature at admission, the SF ratio, and the LUS score, with a Nagelkerke's *R*^2 ^= 0.458 (*p* < 0.001). The predictive model showed an area under the curve = 0.84 (95% CI, 0.75–0.93, *p* < 0.001).

**Conclusions:**

CPAP failure after LISA is still common in extremely preterm infants, leading to an increase in death or disability. Clinicians must acknowledge the main risk factors of CPAP failure to choose wisely the right patient and the best technique. LUS and the SF ratio at admission can be useful when making these decisions.

## Introduction

Respiratory distress syndrome (RDS) remains the leading cause of morbidity and mortality in preterm infants (1, 2). Surfactant therapy and non-invasive ventilation is an effective treatment for improving outcomes in this population (3). Nonetheless, determining the optimal management strategy deserves careful consideration of individual patient factors.

Based on large clinical trials, less-invasive surfactant administration (LISA) has been accepted as the preferred method of surfactant administration for spontaneously breathing preterm infants on continuous positive airway pressure (CPAP), with better outcomes than surfactant administration via an endotracheal tube (ETT) (3–5).

However, CPAP failure after LISA, defined as the need for intubation in the first 72 h after birth, is still frequent, especially in extremely preterm infants. In several studies, patients with CPAP failure have shown worse outcomes in terms of pneumothorax, bronchopulmonary dysplasia (BPD), and death than those managed successfully on CPAP (6–10).

In addition, LISA may result in delayed surfactant administration due to the need for a longer stabilization than administration via an ETT in the neonatal intensive care unit (NICU) (6). Whether mid- and long-term outcomes of patients who undergo CPAP failure after LISA are comparable with those intubated before surfactant administration remains unclear. Moreover, studies aimed at identifying which patients would benefit from surfactant administration via an ETT and which are suitable candidates for LISA are limited and observational (6, 8, 11). A judicious selection of patients among those at higher risk of CPAP failure after LISA could improve outcomes and avoid repeated procedures and economic waste.

The integration of lung ultrasound (LUS) with appropriate training has been demonstrated to be useful in predicting the need for surfactant therapy even better than FiO_2_ or chest X-ray (12). LUS is being widely accepted in neonatal units as a standard of care (13); however, no previous publications have assessed LUS accuracy in identifying patients at a higher risk of CPAP failure after LISA. In this study, we aimed to assess the outcomes of preterm infants who received surfactant for RDS, comparing an ETT and LISA, and to evaluate the risk factors associated with CPAP failure after LISA.

## Material and methods

### Patients

This was an observational study carried out over 6 years (2017–2022) in two NICUs in Barcelona, Spain (Hospital Clinic Barcelona and Hospital Sant Joan de Déu). Inborn preterm infants born between 23^0^ and 33^6^ weeks gestational age (GA) who received surfactant were included. Exclusion criteria were major congenital malformations. Patient selection adhered to the Declaration of Helsinki and applicable local regulatory requirements after approval from the Institutional Review Boards (reference number HCB/2022/1011).

### Stabilization in the delivery room and respiratory management

Delivery room (DR) interventions in preterm infants born less than 30 weeks GA included delayed cord clamping and plastic wrap. Intermittent positive pressure using a face mask with a positive end-expiratory pressure (PEEP) of 6–8 cmH_2_O and peak inspiratory pressure of 20–25 cmH_2_O was started in apneic or bradycardic infants. Spontaneously breathing infants were stabilized with a humidified CPAP and RAM cannula (PEEP 7–9 cmH_2_O). The initial FiO_2_ was 0.30, which was then titrated to achieve an oxygen saturation of 90% at 10 min of life based on preductal pulse oximetry (14). At admission, CPAP was continued via a nasal mask with a PEEP between 6 and 8 cmH_2_O. The oxygen saturation target range was 90%–95%. Intravenous caffeine and parenteral nutrition were also started at admission. Surfactant therapy (200 mg/kg; Curosurf®, Chiesi Pharmaceuticals, Parma, Italy) was administered according to the European guidelines (3), using the LISA method in infants with CPAP and stable vital signs. According to local guidelines, patients were not suitable for LISA if they had hemodynamic instability, an air leak, FiO_2_ requirements >0.60, an altered acid-base test (pH < 7.00, pCO_2_ > 65 mmHg, base excess (BE) < −10), or an LUS score ≥15 (if LUS was performed at admission). Those patients received surfactant via an ETT. A second dose of surfactant (100 mg/kg) could be administered if needed after 6 h. We used mechanical ventilation (MV) with volume-guaranteed (4–5 ml/kg) and high-frequency oscillatory ventilation with volume-guaranteed in case of peak inspiratory pressures >22–25 cmH_2_O depending on GA and birth weight. Echocardiographic screening for patent ductus arteriosus (PDA) was performed at the discretion of the neonatologist in charge of the patient in the first week of life. Respiratory management was carried out according to local protocols.

### LISA procedure

LISA was the preferred method for all those preterm infants with spontaneous breathing but the final decision on the technique of surfactant delivery rested with the attending physician, as did the decision for pharmacologic sedation or atropine administration for the procedure.

The following steps are contemplated in our guidelines for the LISA procedure:

1. Apply a nasal mask and CPAP pressure optimization (6–8 cm H_2_O).
2. Administration of glucose perfusion and caffeine base (10 mg/kg).
3. Perform an imaging test, either an LUS or chest X-ray.
4. Patient preparation: restraint by one caregiver, quiet environment, temperature, light and noise control, colostrum or sucrose, and pacifier.
5. Material preparation: catheter (Surfcath®, Vygon, Valencia, Spain, or Lisacath®, Chiesi), conventional laryngoscope or videolaryngoscope, Steri-Strip to mark on the catheter the estimated distance from the mouth to the larynx (5.5 cm + patient weight), 5–10 ml syringe, loading needle, gastric tube, and suction tube.
6. Pre-warming of surfactant.
7. Optimize patient comfort and individualize the need for sedation or atropine.
8. Monitoring: ensure adequate vital signs and temperature control during the procedure.
9. Use direct laryngoscopy with a standard blade or indirect videolaryngoscopy with a hyperangulated blade (GlideScope®, Verathon Inc, Bothell, WA, USA). After catheter placement, remove the laryngoscope. Administration of surfactant within 1–2 min. Subsequently, administration of 2 cm of air. Remove the catheter.
10. Post-procedure patient accommodation and optimize non-invasive ventilation.

### Lung ultrasound

Bedside LUS was performed at admission (between 60 and 120 min of life) using a linear probe with warmed gel. The complete LUS protocol has been described previously (15). Patients were in the supine position and each lung was divided into three areas defined by the midclavicular line (anterior), the anterior axillary line (lateral), and the posterior axillary line (posterior) and through longitudinal orientation, and LUS clips were recorded (16). In patients stabilized with CPAP, an LUS was performed to guide surfactant administration, and in patients who underwent endotracheal intubation in the DR, the LUS was used to rule out pneumothorax or atelectasis before surfactant treatment.

### Outcomes and definitions

Demographic data and main outcomes during admission were collected. Respiratory variables, days of MV, days of oxygen, oxygen saturation, FiO_2_, the saturation/fraction of inspired oxygen (SF) ratio, type of respiratory support, mean airway pressure, the time of surfactant administration, the need for postnatal steroids, and the duration of respiratory support before discharge were also documented. CPAP failure after LISA was defined as the need for MV in the first 72 h of life. BPD was defined according to the 2001 Workshop (17). Intraventricular hemorrhage (IVH) was graded according to Papile's classification (18), necrotizing enterocolitis (NEC) was defined using Bell's classification (19), and retinopathy of prematurity (ROP) was classified according to the International Committee for Classification of ROP (20).

### Statistical analysis

Normally distributed variables are presented as mean and standard deviation, whereas non-normally distributed variables are presented as the median and interquartile range (25th–75th centile). Univariate analysis (chi-squared test or Fisher's exact test) was performed for categorical comparisons, and Student’s *t*-test or a Mann–Whitney test were used with continuous variables. Multivariate analysis was used to assess the association between CPAP failure after LISA and the main outcomes (death, BPD, and severe IVH) adjusted for GA. Logistic regression with odds ratios and its 95% confidence interval (95% CI) was performed for each predictor of CPAP failure after LISA, and Nagelkerke's coefficient *R*^2^ was calculated to assess the predictive model including the best predictors (those with *p* < 0.10 in the univariate analysis). The area under the receiver operating characteristic (ROC) curve (and its 95% CI) for the final predictive model was also assessed. Bilateral *p*-values inferior to 0.05 were accepted as statistically significant. Statistical analysis was performed using SPSS 22.0 (IBM Corporation, New York, NY, USA).

## Results

**Figure 1:**
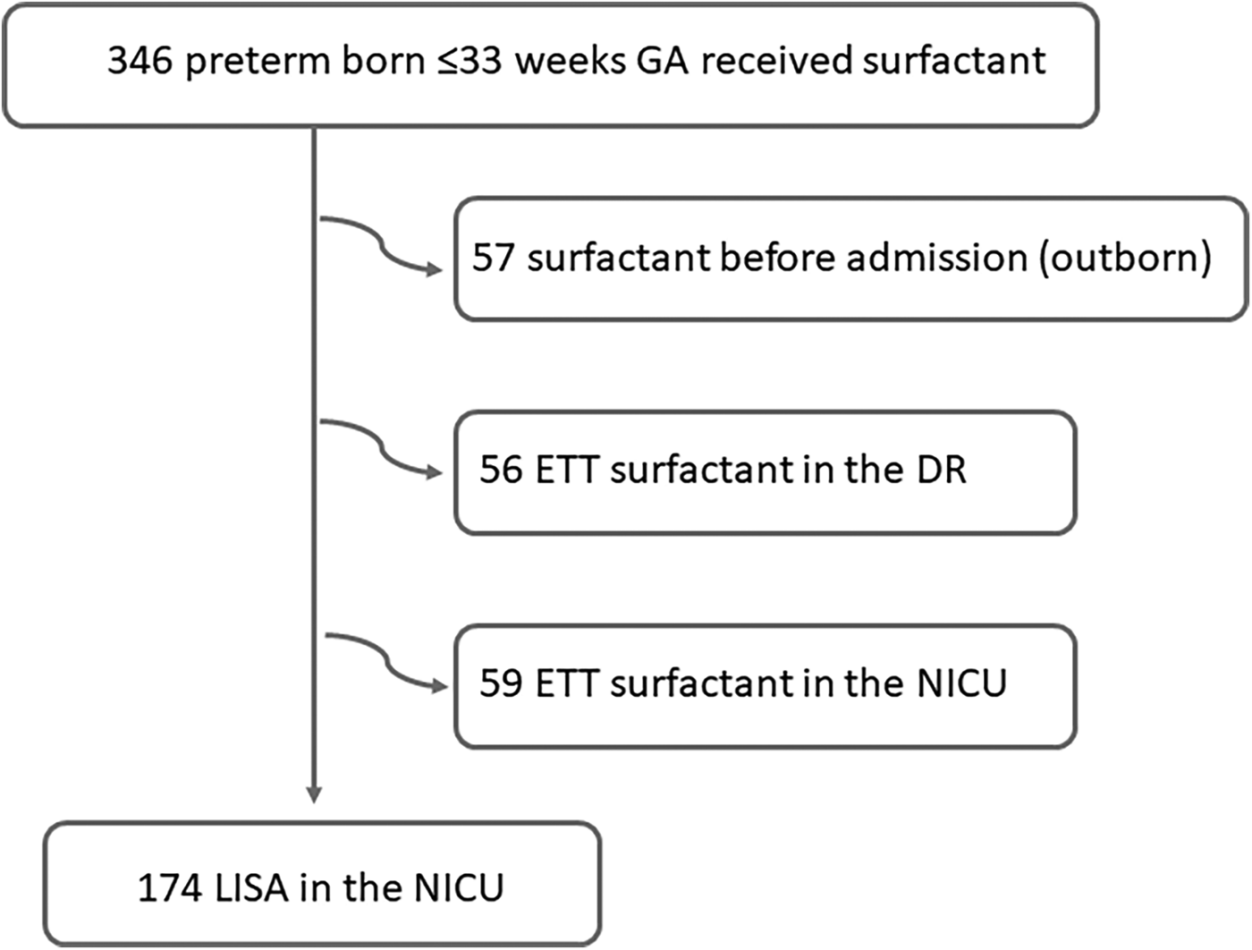
Flowchart of the study population.

Over the study period, 289 inborn preterm infants with a GA between 23^0^ and 33^6^ weeks received surfactant; 174 with the LISA method (60.2%) and 115 via an ETT, as shown in Figure 1. The median administration of surfactant in the whole cohort was 3 h [2–5].

In Table 1, we summarize the study population's baseline characteristics according to the surfactant administration method. Patients who received surfactant after intubation in the DR were more immature and exhibited worse outcomes (IVH, BPD, and death).

**Table 1:** Baseline characteristics and main outcomes of the study population according to surfactant administration.

	ETT in DR (*n* = 56)	ETT in the NICU (*n* = 59)	LISA in the NICU (*n* = 174)	*p*-value
Perinatal data				
GA (weeks)	26.1 ± 2.8	27.5 ± 3.0	28.9 ± 2.5	<0.001
Birth weight (g)	831 ± 434	1,021 ± 469	1,197 ± 468	<0.001
IUGR	13 (24.1)	15 (25.4)	47 (27.5)	0.868
Male sex	29 (51.8)	33 (55.9)	104 (59.8)	0.556
Antenatal steroids (≥2 doses)	30 (53.6)	46 (78.0)	107 (61.5)	0.018
Oligoamnios	16 (29.1)	14 (24.1)	18 (10.3)	0.001
Multiple gestation	11 (20.4)	17 (31.5)	47 (29.9)	0.341
Cesarean section	28 (50.0)	38 (64.4)	124 (71.3)	0.014
Apgar 5 min	5.5 (4–7)	8 (7–9)	8 (7–9)	<0.001
CRIB II score	12 (8–14)	10 (7–13)	7 (4–10)	<0.001
Temperature (°C) at admission	36.3 ± 0.9	36.6 ± 0.7	36.8 ± 0.6	0.002
pH at admission	7.18 ± 0.15	7.18 ± 0.12	7.24 ± 0.08	0.002
pCO_2_ at admission	54 ± 23	60 ± 20	52 ± 11	0.028
CRP at admission (mg/dl)	1.05 ± 2.8	0.40 ± 0.6	0.32 ± 0.9	0.034
Hematocrit at admission (g/L)	42 ± 7	46 ± 9	48 ± 7	<0.001
Surfactant (h)	0.5 (0–1)	3.0 (2–6.1)	4.0 (3–6.8)	<0.001
Neonatal outcomes				
Early-onset sepsis	11 (19.6)	8 (14.3)	15 (8.6)	0.071
Pneumothorax	5 (9.1)	6 (10.7)	18 (10.3)	0.954
PDA	31 (64.6)	32 (60.4)	75 (43.5)	0.009
Postnatal steroids	9 (23.1)	8 (22.2)	8 (7.3)	0.012
Severe IVH	16 (44.4)	8 (22.9)	8 (9.8)	<0.001
Severe ROP	7 (18.4)	8 (18.2)	13 (8.2)	0.070
Moderate to severe BPD	18 (47.4)	20 (42.6)	27 (17.2)	<0.001
Home oxygen therapy	12 (34.3)	12 (25.5)	13 (8.3)	<0.001
Death	19 (33.9)	13 (22.0)	14 (8.0)	<0.001
Length of stay (days)	92 ± 38	82 ± 43	63 ± 35	<0.001

BPD, bronchopulmonary dysplasia; CRP, C-reactive protein; DR, delivery room; ETT, endotracheal tube; GA, gestational age; IUGR, intrauterine growth restriction; IVH, intraventricular hemorrhage; LISA, less-invasive surfactant administration; NICU, neonatal intensive care unit; PDA, patent ductus arteriosus; ROP, retinopathy of prematurity.

Values are expressed in numbers (%) and mean ± standard deviation or median (25th–75th centile).

Of the 233 patients who were stabilized with CPAP in the DR and admitted to the NICU, 59 were intubated and received surfactant via an ETT and 174 received surfactant with LISA (Table 2). One-third received two or more doses of surfactant, with no differences according to the administration technique.

**Table 2:** Respiratory status in preterm infants stabilized with CPAP who received surfactant via LISA vs. ETT in the NICU.

	ETT in the NICU (*n* = 59)	LISA in the NICU (*n* = 174)	*p*-value
SF ratio at admission	200 (147–356)	298 (232–350)	0.194
LUS score^a^	12 (11–15)	11 (9.3–12)	0.013
Caffeine before surfactant	35 (62.5)	135 (80.4)	0.007
FiO_2_ just before surfactant	0.50 (0.39–1)	0.40 (0.35–0.50)	0.001
FiO_2_ 1 h after surfactant	0.25 (0.21–0.31)	0.25 (0.21–0.30)	0.210
Decrease in FiO_2_ after surfactant	0.19 (0.09–0.40)	0.14 (0.09–0.20)	0.033
Mechanical ventilation (h)	120 (30–480)	0 (0–72)	<0.001
Antibiotic (days)	6.5 (3–16.3)	3 (2–7.5)	0.011
Parenteral nutrition (days)	10 (5.8–15.8)	9 (6–12.5)	0.179
Treated PDA	18 (54.5)	36 (48.0)	0.531
Oxygen (days)	27 (3–75.5)	7 (2–40.3)	0.001

DR, delivery room; ETT, endotracheal tube; FiO_2_, fraction of inspired oxygen; LISA, less-invasive surfactant administration; LUS, lung ultrasound; NICU, neonatal intensive care unit; PDA, patent ductus arteriosus; SF, saturation/fraction of inspired oxygen.

Values are expressed in numbers (%) and mean ± standard deviation or median (25th–75th centile).

^a^ Performed at 60–120 min.

Patients undergoing MV, regardless of when they were intubated (*n* = 168), exhibited a higher risk, when adjusting for GA, of severe IVH, with an adjusted odds ratio (aOR) = 9.7 (95% CI, 1.2–80.8, *p* = 0.037); moderate to severe BPD, with an aOR = 5.8 (95% CI, 2.5–13.6, *p* < 0.001); and home oxygen therapy, with an aOR = 6.3 (95% CI, 1.7–22.7, *p* = 0.005). We found a rate of pneumothorax of 10.2%, with significant differences according to GA (18.5% in <28 weeks GA vs. 5.5% in ≥28 weeks; *p* = 0.007).

LISA was attempted in 180 patients, failing in 6 (3.3%), who were intubated before performing the LISA procedure. Sedation for LISA was used in 14.2% of cases and 26 patients (9.4%) were premedicated with atropine. Ten patients presented a decrease in saturation of <80% and bradycardia (HR < 100 bpm) during LISA (2.6%).

LUS scores were recorded in 94 patients (56%) before LISA. Compared with surfactant via an ETT in the NICU, the LISA method was also associated with less death or BPD, adjusted for GA, with an aOR = 0.37 (95% CI, 0.18–0.74, *p* = 0.006).

**Figure 2:**
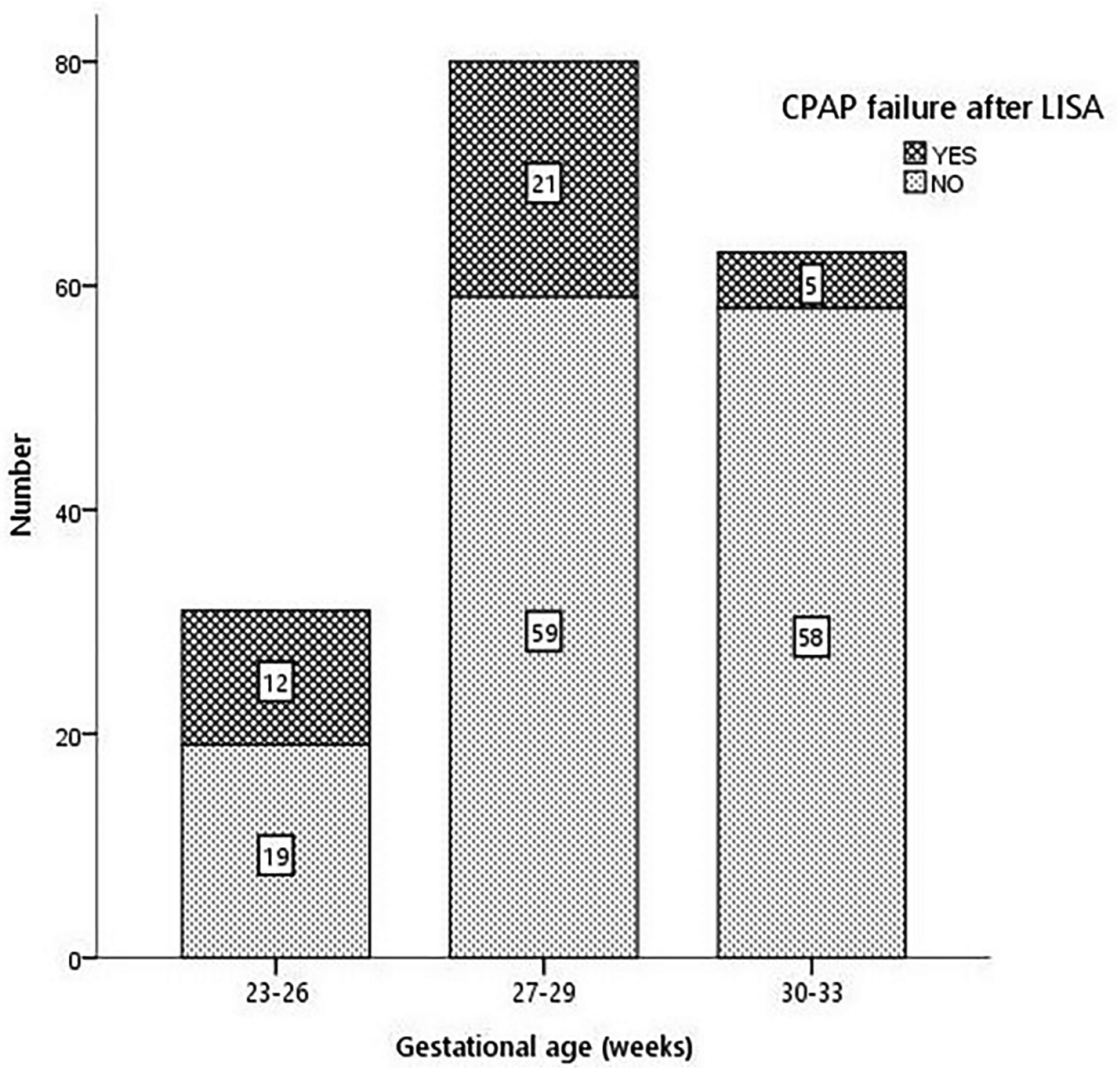
CPAP failure after LISA according to gestational age.

The rate of CPAP failure after LISA was 21.8% (38 patients) and was inversely proportional to GA (38.7% in 23–26 weeks, 26.3% in 27–30 weeks, and 7.9% in 30–33 weeks, *p* < 0.001), as shown in Figure 2. Infants failing CPAP after LISA had higher rates of pneumothorax (36.8% vs. 2.9%, *p* < 0.001) and early-onset sepsis (18.4% vs. 2.9%, *p* = 0.015).

**Figure 3:**
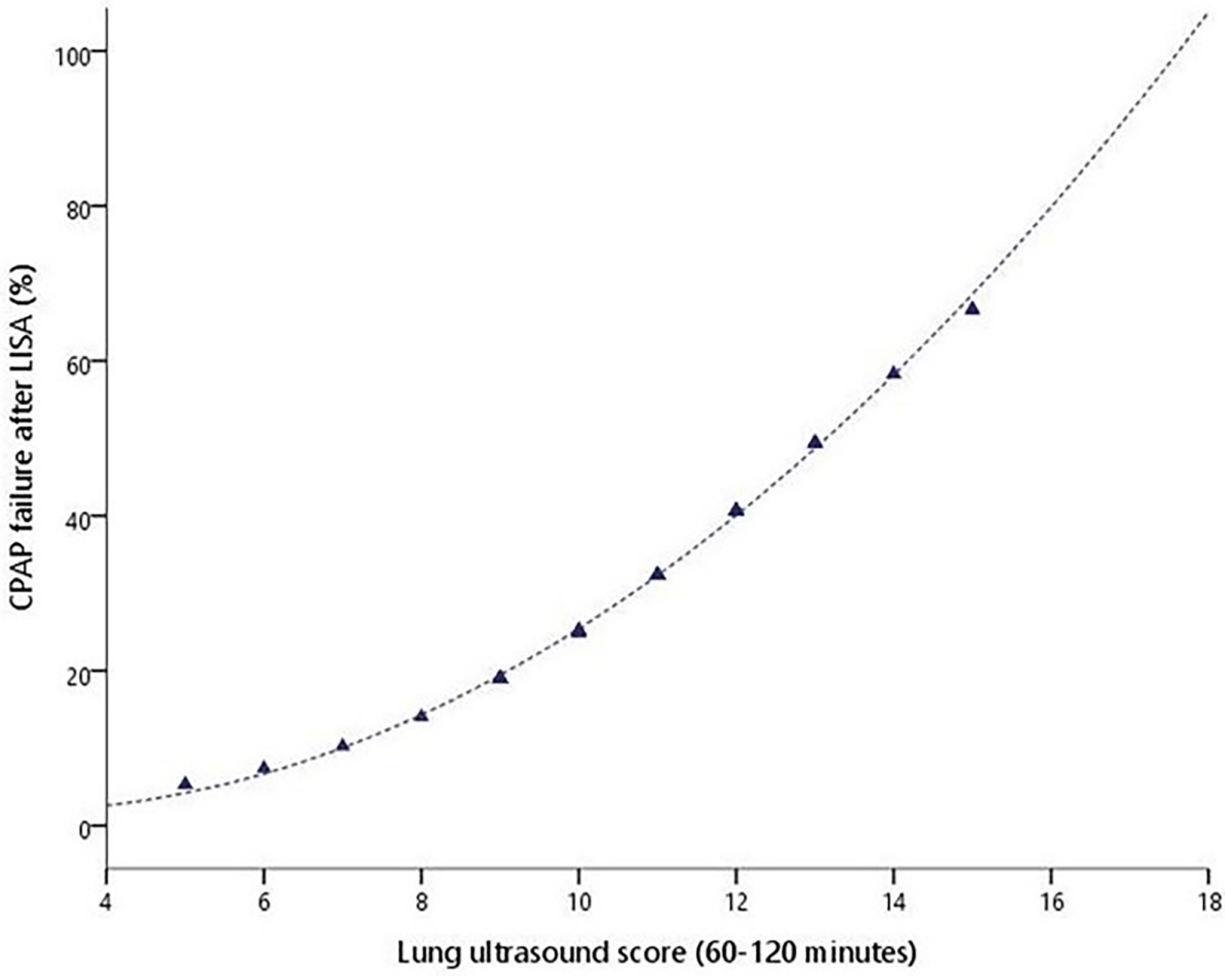
CPAP failure after LISA probability (%) according to the LUS score obtained between 1 and 2 h of life.

The demographic characteristics, main outcomes, and risk factors of CPAP failure after LISA are represented in Table 3. The SF ratio and LUS score recorded at 60–120 min of life were good predictors of CPAP failure after LISA (Figure 3). Table 4 summarizes the best predictors of CPAP failure after LISA. We found that the model including the early predictors of CPAP failure after LISA had a Nagelkerke's coefficient *R*^2^ of 0.458 and an area under the curve (AUC) = 0.84 (95% CI, 0.75–0.93, *p* < 0.001).

**Table 3:** Demographics and main outcomes according to CPAP failure after LISA.

	CPAP failure after LISA		*p*-value
	Yes (*n* = 38)	No (*n* = 136)	
Perinatal data			
GA (weeks)	27.5 ± 2.1	29.3 ± 2.4	<0.001
Birth weight (g)	923 ± 369	1,273 ± 465	<0.001
IUGR	17 (44.7)	30 (22.6)	0.007
Male sex	28 (73.7)	76 (55.9)	0.048
Antenatal steroids (≥2 doses)	28 (73.7)	79 (58.1)	0.081
Oligoamnios	7 (18.4)	11 (8.1)	0.064
Temperature at admission (°C)	36.4 ± 0.6	36.9 ± 0.6	<0.001
Respiratory status after birth and surfactant response			
pH at admission	7.23 ± 0.08	7.24 ± 0.07	0.387
pCO_2_ at admission	50 ± 12	52 ± 11	0.529
SF ratio^a^	270 [188–300]	303 [250–360]	0.005
LUS score^a^	12 [10–13]	11 [9–12]	0.218
LUS score >12^a^	6 (28.6)	5 (8.5)	0.022
Surfactant (h)	3 [2–4]	4 [3–9]	0.004
FiO_2_ before surfactant	0.45 [0.35–0.71]	0.40 [0.33–0.49]	0.017
FiO_2_ 1 h after surfactant	0.30 [0.25–0.38]	0.23 [0.21–0.27]	0.001
Decrease in FiO_2_ after surfactant	0.10 [0.01–0.20]	0.14 [0.09–0.20]	0.041
Neonatal outcomes			
Early-onset sepsis	7 (18.4)	8 (5.9)	0.015
Pneumothorax	14 (36.8)	4 (2.9)	<0.001
Mechanical ventilation (hours)	132 [72–366]	0 [0–0]	<0.001
Antibiotic (days)	8.5 [2.8–15]	3 [2–7]	0.003
Oxygen (days)	19 [6.8–64]	5 [1–34.5]	0.017
Postnatal steroids	2 (7.7)	6 (7.2)	0.937
Moderate to severe BPD	10 (38.5)	17 (13.0)	0.002
Home oxygen therapy	6 (23.1)	7 (5.4)	0.003
Severe IVH	4 (23.5)	4 (6.2)	0.032
Death	11 (28.9)	3 (2.2)	0.032
Surgical NEC or SIP	3 (10.7)	11 (8.1)	0.659
Death	11 (28.9)	3 (2.2)	0.032

BPD, bronchopulmonary dysplasia; FiO_2_, fraction of inspired oxygen; GA, gestational age; IUGR, intrauterine growth restriction; IVH, intraventricular hemorrhage; LUS, lung ultrasound; NEC, necrotizing enterocolitis; SF, saturation/fraction of inspired oxygen; SIP, spontaneous intestinal perforation.

Values are numbers (%) and mean ± standard deviation or median (25th–75th centile).

^a^ Performed at 60–120 min.

**Table 4:** Early predictors of CPAP failure after LISA in preterm infants.

	OR (95% CI)	*p*-value
Male sex	2.21 (1.00–4.91)	0.05
GA < 28 weeks	3.97 (1.87–8.44)	<0.001
IUGR	2.81 (1.32–5.99)	0.008
Temperature (°C) < 36.5°C at admission	4.99 (2.26–11.02)	<0.001
LUS score >12^a^	6.38 (2.03–20.0)	0.002
SF ratio <300^a^	3.24 (1.47–7.15)	0.004

CI, confidence interval; GA, gestational age; IUGR, intrauterine growth restriction; LISA, less-invasive surfactant administration; LUS, lung ultrasound; OR, odds ratio; SF, saturation/fraction of inspired oxygen.

^a^ Performed at 60–120 min.

Patients who failed CPAP after LISA exhibited worse outcomes than those with a successful LISA procedure adjusted for GA, such as death with an aOR = 12.0 (95% CI, 3.0–47.8, *p* < 0.001) and moderate to severe BPD with an aOR = 2.9 (95% CI, 1.1–8.0, *p* = 0.035), but not severe IVH (*p* = 0.144).

There were no significant differences in days of MV, days of oxygen, or in the main outcomes in patients failing CPAP after LISA compared with those receiving surfactant via an ETT in the NICU when adjusting for GA: severe IVH (aOR = 1.10, 95% CI, 0.25–4.81, *p* = 0.900), moderate to severe BPD (aOR = 0.85, 95% CI 0.31–2.30, *p* = 0.743), and death (aOR = 1.80, 95% CI, 0.65–5.02, *p* = 0.261).

## Discussion

CPAP failure after LISA is still common in extremely preterm infants, with RDS leading to an increase in death or disability. In the present study, CPAP failure after LISA occurred in one in five patients, with RDS falling within the lower range compared with similar studies (6, 8). Nevertheless, CPAP failure after LISA was significantly related to death (OR = 12.0) and moderate to severe BPD (OR = 2.9) compared with CPAP success after LISA.

Many factors associated with a higher risk of CPAP failure in patients with RDS undergoing LISA have already been identified (4, 6–9, 11). This study adds the value of a predictive model with an AUC = 0.84 (95% CI, 0.75–0.93, *p* < 0.001), with GA, intrauterine growth restriction (IUGR), temperature at admission, the SF ratio, and LUS score between 60 and 120 min being the strongest predictors of CPAP failure and male sex being more frequently associated with CPAP failure. Other conditions such as early-onset sepsis are also related to CPAP failure after LISA, but in most cases are difficult to identify in the first hours of life and therefore cannot be considered when deciding whether or not to perform the LISA procedure.

One of the strengths of our study lies in exploring the role of LUS in predicting CPAP failure after LISA; this study preludes that it is a promising tool for the early identification of patients who would benefit from surfactant administration via an ETT. It should be noted that the NICUs where the study was performed have extensive experience in LUS, using it in some cases to select patients undergoing LISA.

The incidence of CPAP failure after LISA in our study may be attributed to the clinical practice, given the observational design of the study. Consistent with similar investigations, infants undergoing intubation in the DR were more immature and exhibited worse outcomes, indicative of a natural inclination toward initially selecting the most stable patients for non-invasive ventilation. In addition, neonates intubated in the NICU for surfactant administration also displayed lower GA, a greater incidence of oligohydramnios, lower pH at admission, and higher CRIB II scores. Notably, in our study, these patients exhibited higher LUS and a greater oxygen requirement than those undergoing LISA, signifying a secondary active selection process, as delineated in Tables 1, 2.

The central question revolves around whether patients who experience CPAP failure following LISA are at a heightened risk of complications compared with those subjected to receiving surfactant via an ETT. Patients intubated in the NICU received earlier surfactant administration and experienced a more rapid decrease in oxygen need than those undergoing LISA, suggesting a potential delay in surfactant administration and lung recruitment with the LISA procedure. However, upon comparing patients experiencing CPAP failure with those undergoing endotracheal intubation, our study did not find significant differences in the duration of MV, oxygen requirement, or primary outcomes (severe IVH, moderate to severe BPD, or death). Despite our rate of CPAP failure after LISA, the LISA method in the overall cohort was significantly associated with less death or BPD compared with endotracheal intubation (OR = 0.37).

Considering these findings, LISA should be the preferable choice in stable patients according to guidelines (3). However, it appears advisable to reconsider LISA in those patients with RDS when presenting a lower GA at birth (<27 weeks), an SF ratio <250, and an LUS >12 at 60–120 min, particularly in cases of IUGR and when the admission temperature is below 36°C. These cases could benefit from other surfactant administration methods such as INtubation-SURfactant-Extubation (INSURE) (21) or INtubate-RECruit-SURfactant-Extubate (IN-REC-SUR-E) (22) to limit MV exposure. Nonetheless, it seems crucial to focus on improving the LISA method to prevent lung injury caused by MV, even if it is only applied for a short duration. These strategies must include improving lung recruitment before and after surfactant administration (23), avoiding hypothermia in the DR, increasing surfactant dosage to account for the usual surfactant glottic reflux (24), and adding anti-inflammatory drugs to the surfactant, especially when perinatal inflammation has been detected (elevated levels of interleukin-6 in the amniotic fluid, ureaplasma infection, etc.) (25, 26).

Several limitations must be acknowledged. First, the observational design of the study and the incomplete LUS evaluation before LISA (LUS scores were only available in 56% of the patients) prevented definitive conclusions and a comprehensive assessment of the role of LUS in deciding the best method for delivering surfactant. In the future, ongoing multicentric randomized controlled trials could better address this question (27). In addition, the GA of the study population ranged from 23 to 33 weeks of gestation, which introduced heterogenicity. Exploring the main factors related to CPAP failure after LISA stratified by GA would have been interesting if we had a larger sample size. Moreover, the predictive model's ability to predict CPAP failure after LISA was limited, accounting for only 46% of the variance. This may be attributed to unexplored variables, such as specific reasons for CPAP failure, or predisposing factors, including intra-amniotic inflammation (25), diaphragmatic dysfunction (28), and vascular remodeling (29). The strategies for avoiding LISA failure have not been considered in our study but certainly deserve further attention in randomized controlled trials. In addition, BPD has important limitations as the primary outcome for evaluating respiratory strategies. Other endpoints, assessed by lung imaging and respiratory function tests, could provide a better discrimination of their medium- or long-term effects (30–32).

To conclude, according to guidelines, LISA on CPAP continues to yield better outcomes than endotracheal intubation, even when considering the risk of CPAP failure after LISA. Clinicians must acknowledge the main risk factors for CPAP failure after LISA to judiciously select the appropriate patient and technique when treating RDS with surfactant. LUS and the SF ratio at admission may serve as valuable tools in this decision-making process. Clinical trials are needed to address this question.

## Data Availability

The raw data supporting the conclusions of this article will be made available by the authors, without undue reservation.
